# The role of platelet gel in osteoarticular injuries of young and old patients

**DOI:** 10.1186/s12979-014-0021-9

**Published:** 2014-12-02

**Authors:** Claudia Rizzo, Roberta Vetro, Angelo Vetro, Roberto Mantia, Angelo Iovane, Marco Di Gesù, Sonya Vasto, Laura Di Noto, Giuseppina Mazzola, Calogero Caruso

**Affiliations:** Unit of Transfusion Medicine, University Hospital “Paolo Giaccone”, Palermo, Italy; Department of Pathobiology and Medical and Forensic Biotechnologies, University of Palermo, Palermo, Italy; Mantia Medical Center, Palermo, Italy; Department of Legal Sciences, Society and Sport, Palermo, Italy; Department of Science and Biological, Chemical and Pharmaceutical Technologies, University of Palermo, Palermo, Italy; National Center for Research, Institute of Biomedicine and Molecular Immunology, Palermo, Italy

**Keywords:** Ageing, Inflammation, Osteoarticular injuries, Osteoarthritis, Regenerative medicine

## Abstract

**Background:**

The use of autologous platelet gel in orthopedics is effective in accelerating the healing process of osteochondral, muscle, tendon and ligament lesions. The aim of our study was to verify whether the variability in response to infiltration with platelet gel was dependent on the underlying disease treated, sex and age of the patients. During four years, 140 patients have been treated for musculoskeletal injuries by infiltration of gel platelet and lysate platelet obtained from autologous thrombin, with echo-ultrasound guided. The response to treatment was assessed at different time points T0, T1, T2 with respect to pain estimation (VAS), joint mobility (ROM scale) and echo-ultrasound evaluation. This data collection has allowed classifying the response to treated lesions in three categories: NR (no response), PR (partial response), CR (complete response).

**Results:**

The data here reported showed that the ability to physical recovery response is evident in tendon injuries, while the large joints injuries gave a poor response. Almost all patients showed a significant pain relief after the first infiltration, but in terms of echo-ultrasound evaluation and tissue repair, only the muscle and tendon injuries showed hyperechoic areas, signs or evidences of repair. Concerning the correlation between response to infiltration with platelet gel and gender/age of the patients, the clinical results appear not influenced by the age and the gender of the patient.

**Discussion:**

Our data indicate that, pain relief and ability to physical recovery of muscles, tendons and ligaments depend on tissue repair clearly visible by echo ultrasound evaluation. On the other hand tissue repair seems not occur in the large joints (hip and knee) where arthritis and /or corrosion of articular cartilage cannot be repaired and the only relief is exclusively linked to the reduction of periarticular inflammation (reduction of the inflammatory leakage and signs).

## Background

The idea of using blood products for not strictly transfusional purposes dates back to the early 70s, when for the first time, fibrin glue was produced and used to accelerate tissue repair during surgery. In the 80s, for the first time, David Knighton developed a technique of in vitro stimulation of platelets by thrombin solutions, which allowed the collection of a rich growth factors supernatant, that suitably purified, was applied topically under the form of gel. It was clear that this gel formulation was able to stimulate the repair of skin ulcers and triggered and accelerated the processes of tissue regeneration. The paramount role of platelets as agents of tissue regeneration, has suggested this application in a variety of clinical settings and for aesthetic purpose [1].

Platelet are not only the protagonists of the haemostatic process, but plays also a key role in the inflammatory process (due to high concentrations of pro-inflammatory and immune-modulatory cytokines), in the antimicrobial defence (since the α- granules are rich in “protein microbicide platelet”, chemokines -CXCL4, thymosin-β4, derivatives of CXCL7- PBP, CTAP - III, NAP- 2 and CCL5 -6 and complement proteins), in cell replication (mitogenesis), in angiogenesis and, last but not least, actively modulate tissue regeneration. These activities are possible because platelet precursors, megakaryocytes, actively produce growth factors (VEGF, PDGF, FGF, EGF, HGF, IGF) responsible for endothelial and fibroblasts cells permeability, recruitment and proliferation [2-6].

Thus the use of autologous platelet gel in orthopaedics could be an effective therapy in accelerating the healing process not only for bone and osteochondral lesions, but also for muscle, tendons and joints diseases. Currently, the literature data are conflicting regarding the efficacy of this treatment. Several studies show that in reconstructive orthopaedic surgery, the application of platelet gel combined with the bone matrix apposition, confirmed the ability of platelet growth factors to determine bone regeneration, and provide the regenerative stimulus to autologous bone used as a filler and source of osteoblasts [7-9].

In addition, the application of platelet gel in the treatment of traumatic tendon lesions showed significant feedback not only for the tissue regenerative activity, but also for anti-inflammatory and analgesic effect [10,11]. Moreover several data showed platelet gel used as a therapeutic choice in the treatment of epicondylitis, of plantar fasciitis and Achilles tendon injuries with healing delayed. This treatment has been also used in inflammatory hip disease, in chondropathies of the knee, in ankle, in hallux rigidus and in acute and chronic muscle injury treatment [12-15].

However, despite the favourable results obtained in several case series, results from randomized clinical trials are controversial [16-19].

So, the aim of our study was to verify whether the variability in response to infiltration with platelet gel was dependent on the underlying disease treated and sex and age of the patients. Therefore, we selected a group of patients (divided by sex, age and osteoarticular lesions) that have not been treated surgically or with other infiltrating application on the site of injury and we proposed to all the same standard-protocol for the production of platelet gel. We excluded patients with systemic diseases involving osteoarticular system (i.e. autoimmune diseases).

## Results

**Figure 1:**
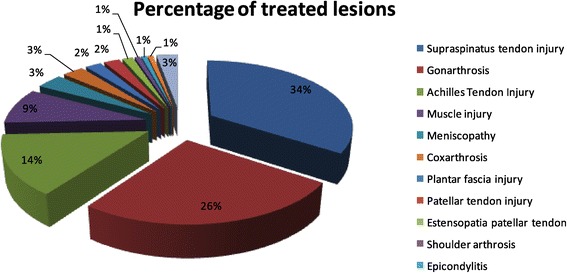
**Percentage of treated lesions in the sample.**

**Figure 2:**
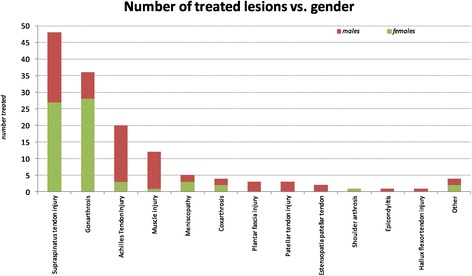
**Distribution of osteoarticular diseases respect to gender.**

From a first glance of the sample population, the pathologies mostly treated with platelet gel are injuries of the supraspinatus tendon, the gonarthritis and injuries of the Achilles tendon (Figure 1). There are differences in diseases distribution especially concerning gender: the female population seems more affected by lesions of the large joints (coxarthritis and gonarthritis) and the supraspinatus tendon while other clinical conditions seems to affect more the male population (Figure 2).

**Figure 3:**
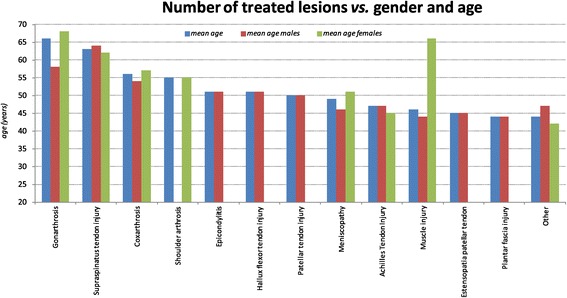
**Osteoarticular lesions, mean age and gender.**

The stratification for age (mean of age 58 years old) showed that arthritic conditions (gonathritis, osteoarthritis, shoulder athritis) and large joints condition (supraspinatus tendon injury) affect a population little older (mean age 60 years), while the ligament and tendon injuries are more frequent in younger subjects (mean age 47 years). When divided by gender and onset of the injury, the female population is often treated for disorders of the knee mostly in middle age with respect to male (68 years females, 58 males), while the tendon and muscle injuries affect male population frequently at younger age (46 years old males, 51 females) (Figure 3).

**Figure 4:**
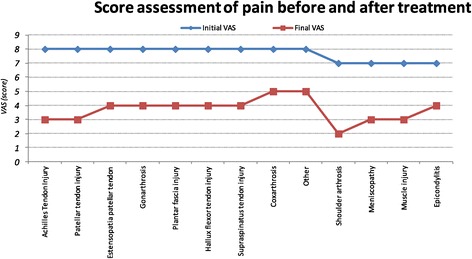
**Trend of the VAS score (mean) in treated diseases.**

Regarding the clinical results, all treated cases in our study, except those with osteoarthritis, (Figure 4) have shown a significant reduction in pain after the first infiltration. These observations appear not influenced by the age and the gender of the patient.

Evidence was achieved only in the study sample with at least 10 treated patients (Table 1), so it hasn’t been achieved a good standardized assessment on the inability to perform physical task on the other few patients treated.

**Table 1:** **Study sample with details of the treated diseases and degree of injury**

**Treated lesions**	**Patients no.**	**No. according to gender (mean age)**		**Degree of injury**	
		**Male**	**Female**		
**Supraspinatus tendon injury**	**49**	21 (64)	28 (62)	Tendinopaty	22%
				Chronic tendinopaty	24%
				partial rupture	31%
				Sub-total rupture	6%
				Total rupture	16%
**Gonarthritis**	**37**	9 (58)	28 (68)		
**Achilles tendon injury**	**20**	17 (47)	3 (45)	Tendinopaty	40%
				Chronic tendinopaty	10%
				Partial rupture	40%
				Sub-total rupture	
				Total rupture	10%
**Muscle injury**	**12**	11 (44)	1 (66)	Distraction muscle	42%
				Muscle tear	8%
				Focal muscle tear	50%
**Meniscopathy**	**5**	2 (46)	3 (51)		
**Coxarthritis**	**3**	1 (54)	2 (57)		
**Plantar fascia injury**	**3**	3 (44)			
**Patellar tendon injury**	**3**	3 (50)			
**Estensopatia patellar tendon**	**2**	2 (45)			
**Shoulder arthritis**	**1**	1 (55)			
**Epicondylitis**	**1**	1 (51)			
**Hallux flexor tendon injury**	**1**	1 (51)			
**Other**	**4**	2 (47)	2 (42)		

**Figure 5:**
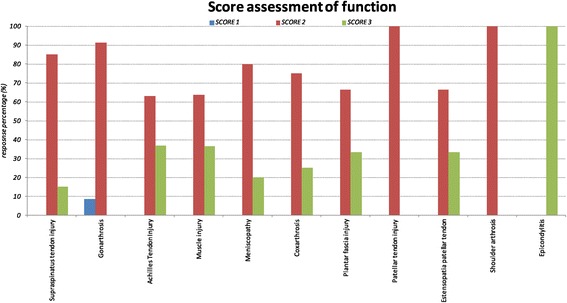
**Functional response (percentage) accordig to functionality score: Score 1 (stable condition than at the beginning of treatment), Score 2 (increase joint range of motion but not full “restitutio ad integrum”), Score 3 (Complete functional recovery).**

However, the data showed complete ability response and fully restoration of joint ability (score 3) in a large part of patients treated for tendon injuries. The treatment of large joints to cure arthritis, instead, gave a poor response in terms of recovery abilities and even almost 10% of the observed gonarthritis did not show any significant result compared to the early status (score 1) (Figure 5).

**Figure 6:**
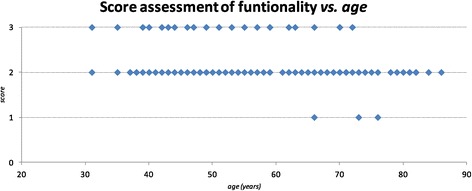
**Functionality (score) related to age of the sample.**

It seems that the success of the treatment (score 2 and score 3) is age independent; only in one case in which the treatment did not gain any effect (score 1) the older age might have been associated to the result (Figure 6).

**Figure 7:**
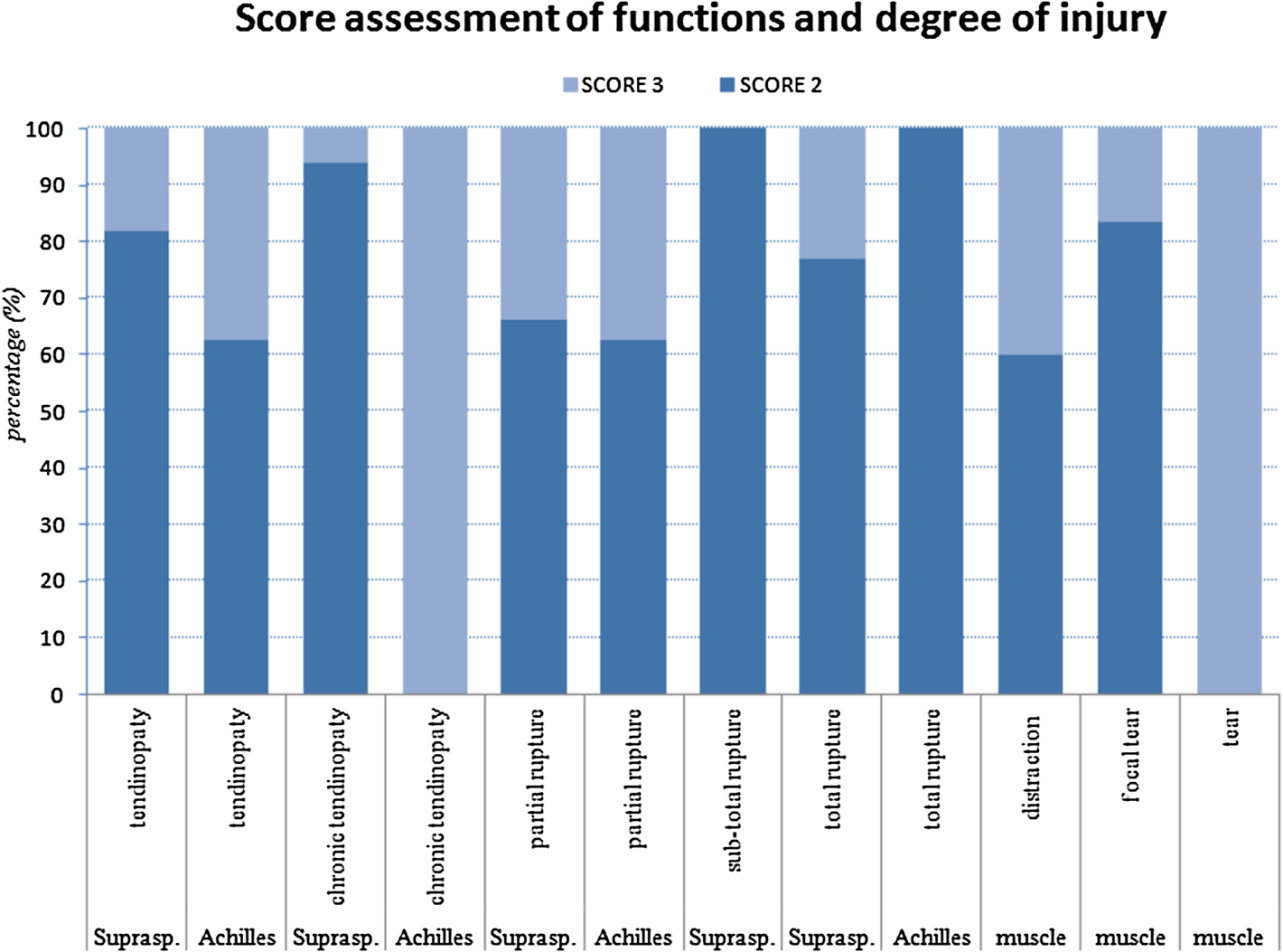
**Functional response (percentage) according to functionality score and degree of injury.**

In addition, the study of the response in relation to the degree of injury (tendinopaty, tendinopaty, chronic partial rupture, sub-total rupture, total rupture) shows that the treatment is independent on the initial lesion (Figure 7).

**Figure 8:**
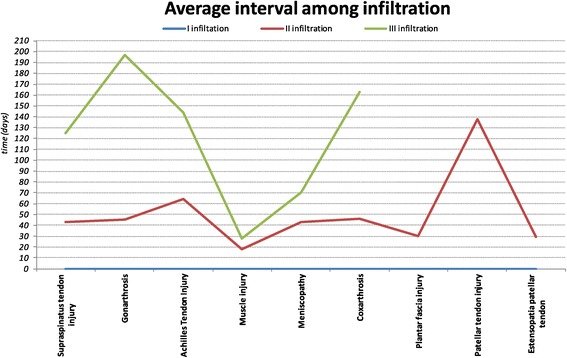
**Average interval (days) among the first, second and third infiltration.**

On the basis of the clinical evaluation after the first infiltration, the patients were given the indication to further infiltration according to time schedule and to the pathology (Figure 8).

**Figure 9:**
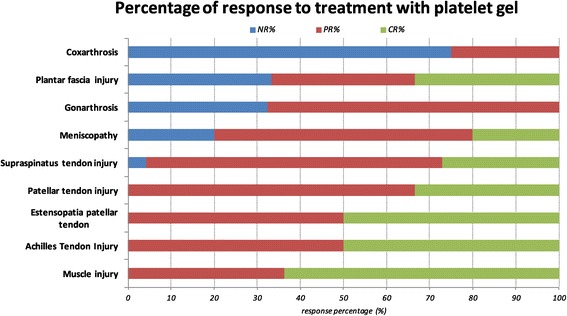
**Response to treatment in osteoarticular diseases treated (percentage).** NR: no response, PR: partial response, CR, complete response.

On average, 102 patients (73% of the population sample) were infiltrated with the second aliquot after a month and a half (48 days) and the third infiltration was performed to 61 patients (44% of the population sample) around five months after the second (158 days). The frequency with which are carried out infiltration is strictly related to the type of lesion: the injuries of tendons and muscles are infiltrated at earlier time schedule than those involving large joints (Figure 9).

The most representative example of tissue repair and recovery ability restoration, is represented by the injury of the Achilles tendon in which after two months of treatment (T2) a complete tissue repair occurred .

Another model of tissue repair is the supraspinatus tendon and muscle injuries: at T1 is already possible to assess the repair of areas with increase of hyperechoic signals.

The lower clinical response is represented by the treatment of osteoarthritis of the large joints (coxarthritis and gonarthritis) where comparing echo ultrasound at T0 and T2. The only sign is referred to the reduction of the inflammatory area and inflammatory leakage but without any sign of cartilage repair.

The infiltration of the cartilage lesion of the knee, such as the lesion of the patellar tendon, instead, showed by diagnostic imaging clear evidence of tissue repair.

## Discussion

The treatment of joint and muscle-tendon injuries by platelet gel is one of the most innovative techniques and generally appreciated by either patients reporting an overall recovery benefits either by clinicians who are studying the effects of tissue repair.

As defined by our protocol, the clinical and instrumental evaluation of response to treatment showed a different distribution of the sample over time (Figure 9):

- NR (No response): 32% of gonarthritis and 75% of coxarthritis treated showed no pain reduction and joint inability and absence of echo-ultrasonographic evidence of tissue repair.
- PR (Partial Response) This group includes the 68% of gonarthritis and 25% of coxarthritis treated. In this category are included most of the tendon lesions treated: like 50% of patellar tendon injury, 69% of supraspinatus tendon injury, 60% of the meniscus, 50% of injuries to the Achilles tendon and 36% of muscle damage.
- CR (Complete Response) This type of response is characteristic of the treatment of tendon injuries (27% of the lesions of the supraspinatus tendon, 33% of injuries to the patellar tendon, 50% of the lesions of the Achilles tendon), the meniscus (20%) and muscle injury (64%). No coxarthritis and gonarthritis issued complete responses.

Regarding to correlation between response to infiltration with platelet gel and gender/age of the patients, the data showed age dependent stratification for treated lesions (arthritic conditions and large joints condition regard a population little older, while the ligament and tendon injuries are more frequent in younger subjects), but the clinical results appear not influenced by the age and the gender of the patient: in particular the success of the treatment is age/gender independent and only in one case in which the treatment did not gain any effect, the older age might have been associated to the result.

Our data highlight that recovery ability and pain relief on muscles, tendons and ligaments depend on tissue repair detectable with echo ultrasonographic evidence. However this seems to not affect the large joints (hip and knee) where the arthritis and/or corrosion of articular cartilage phenomena are not influenced by the infiltration process. Nonetheless, on large joints, the pain relief effect is exclusively linked to the reduction of periarticular inflammation (reduction of inflammatory leakage and inflammatory status).

The rationale of this observation lays on some aspects of the pathology of the large joints and combined with the effect of platelet gel: in fact, the cartilage is a non-vascularized and not innervated tissue, lacking a draining lymphatic system and with limited regenerative ability [20].

The arthritis process is a condition characterized by an unbalanced anabolic and catabolic processes at the articular surface of the synovial joint that result in progressive damage of the cartilage that brings to physical disability [21].

One of the most important inflammatory mediators involved in the arthritic process is represented by IL-1β, capable of inducing the production of proteases and able to inhibit the formation of extracellular matrix. A recent study [22] on in vitro cell cultures of human chondrocytes, has highlighted the role of IL-1β which plays a key role in the pathogenesis of osteoarthritis since determines significant changes in cytokine and chemokines genes expression involved in inflammation and matrix degradation. IL-1β, that exerts its effect through a broad spectrum of signals cascade, is activated by NF-kB (nuclear factor kappa B) and it in turn enhances its translocation into the nucleus [23].

Normally NF-kB is, in fact, located in the cytosol bound to an inhibitory protein and once activated it translocates to the nucleus where it plays in the regulation of more than 150 regulatory genes involved in apoptosis, inflammation, and in immune response [24].

The degranulation of the platelet gel acts on IL- 1β mostly by the inhibition of translocation NF-kB into the nucleus of chondrocytes. Many drugs act as inhibitors of NF-kB including as glucocorticoids, so platelet gel could be used to balance the anabolic/ catabolic effect present in bone and joint disease [25].

Another important aspect, concerns the effect of platelet gel on the production of nitric oxide (NO).

In osteoarthritic disease IL-1β, as well as activates NF-kB, determines increased production of NO. The excess of NO either inhibits the synthesis of collagen and glycosaminoglycans, either induces apoptosis of chondrocytes and the production of metalloproteases [26]. The control of this mechanism is connected to TGF-β (platelet gel contained) which promotes the deposition of collagen and fibroblast proliferation and to the inhibitor role of NF-kB which, in turn, reduces the described effects of IL-1β.

The platelet gel therefore inhibits the IL-1β effects in osteoarthritic chondrocytes, so determining a substantially anti-inflammatory and analgesic effect. Thus, the application of platelet gel on osteoarthritis lesions can improve the quality of the synovial fluid through the induction of endogenous secretion of hyaluronic acid by the synovial cells [27].

However different considerations must be applied to the ligament and tendon injuries in which the platelet gel has the ability to deeply promote tissue regeneration.

The natural history of a pull or a bruise muscle, determines the formation of an hematoma (as a consequence of vascular destruction) that contains about 94% of red blood cells, less than 1% of leukocytes and a small part of platelets (approximately 4%) therefore representing inadequate environment to provide a stimulus for tissue regeneration. The rational use of platelet gel consists in the possibility of bringing to the lesion a large concentration of platelets and released growth factors that directly or indirectly promote tissue repair by inducing myogenesis and supporting muscle contraction [28,29].

The tendon cells (tenocytes and tenoblast) have a central role in repairing and maintaining extracellular matrix integrity by new protein and degrading enzyme synthesis. These processes underlie the turnover, remodeling and the gradual transformation of extracellular matrix that usually occur before the tendon rupture. The activity of tendon cells are influenced by cytokines and growth factors released by platelet gel (especially VEGF and HGF) that act in a paracrine manner [29].

The initial phase of process repair starts with the vascular formation that seems playing a key role in the “restitutio ad integrum” of the lesion followed by tenocytes and fybroblast proliferation [30]. The GFs contained in the platelet gel (TGF- β, PDGF, VEGF, EGF, HGF) interacting with specific receptors located on the membranes of target cells activate intracellular signal transduction pathways and induce the synthesis of proteins involved in angiogenesis and in the formation of extracellular matrix.

Furthermore, the platelet gel contains adhesion molecules (such as fibrin, fibronectin, vitronectin, thrombospondin, osteocalcin and osteonectin) that permits cell activation and movement of the precursor cells at the site of lesion [28].

All this process can repair connective and/ or bone tissue by increasing fibroblasts activates macrophages and stimulates new vessels formation which restore blood flow allowing nutrients and oxygen perfusion in order successfully repair the tissue [4-6].

Given these properties, platelet gel is crucial in the tendons, muscles and ligaments repair, acting on the regenerative stimulus.

Accordingly, in literature [31] it has been demonstrated that the use of platelet gel in the treatment of “tennis elbow” gives therapeutic advantages in terms of pain relief and ability recovery. A comparative case–control study on patients infiltrated with platelet gel and patients infiltrated with cortisone showed that treatment with cortisone quickly gives effect but quickly enough it disappears. On the contrary the benefit of platelet gel treatment slowly increases and tend to be maintained over time [32].

The same result is confirmed by the response to treatment of supraspinatus tendon lesions where patients infiltrated with platelet gel acquire better physical ability recovery in terms of motility and pain reduction and this is maintained over time even after six months from the treatment compared to control group [22,33].

Besides that, many aspects of the production and application of platelet gel are fully understood by the scientific community. The definition of the optimal dose, the amount and the time interval of infiltration remains under debate, because it does not exist a single standard protocol of treatment. The preparation and administration of platelet gel is established by clinical needs, type of pathology and the experience of the operator.

## Conclusions

Regenerative medicine can be considered one of the final frontier for the treatment of many diseases, and it represents a new philosophy to approach tissue/ organ damage using the principle of biological regeneration [5]. Platelet gel is a new approach to tissue regeneration and it is becoming a valuable adjunct to promote healing in many procedures, especially in ageing patients [34]. In particular, in our opinion, platelet gel can be considered as an additional and integrative therapy to support conventional treatments for bone, joint and tendon injuries of different etiology because platelet gel therapy often resulted in pain relieve and significant physical ability. Regardless of whether or not the gel platelet can determine a complete tissue repair, it is doubtless that the therapy promotes analgesic effect with at least a reduced intake of anti-inflammatory drugs.

In our series of elderly patients, it seemingly does not work well because they are mainly affected by long lasting osteoarthritis with irreversible damage to cartilage.

From our observation is difficult to dissect if the contribution of the platelet gel depends on the number of infiltrations or other variables related to treatment, but it seems more likely that the response can be highly variable and extremely subjective.

It is clear that the general clinical condition of the patient (obesity, metabolic syndrome, advanced age, neurodegenerative disorders, strenuous physical activity, concomitant orthopedic disorders) might aggravate the osteoarticular pathology and expose the patient to a poor response or risk relapses.

Secondly, it is recognized by the scientific community [25] the individual variability and the capacity to produce GFs and releasing them into the injury site, which obviously causes a different biological inter-individual response.

Finally, the lacking of universally standardized and detailed protocols for the various bone and joint diseases (for both aspects of the production, and for those relating to the application of the gel) can be responsible for the extreme variability in the therapeutic response.

In view of the beneficial effects that gel platelet determines to tendon and joint lesions, we believe that the creation of a multicenter randomized study can increase and expand the clinical records of treated individual in order to refine and standardized certain aspects of this research.

Furthermore, it would be desirable to define a project that allows to correlate the compliance of the patient, the platelet concentration in the platelet gel and the amount of growth factors contained in it to identify any adjustable variables.

In addition, we have to take into account the possibility of a placebo effect since the expectation of a therapeutic result can activate specific brain regions and these same expectations can sometimes be the actual cause of clinical improvement However, a complete assessment of a placebo effect for this kinds of treatment it is very complicate, because like acupuncture studies, should include the study of 3 arms (treatment, sham treatment, open placebo) with the imaging of cerebral area involved [35,36].

### Subjects, materials and methods

#### Sample studied

In four years of activities (October 2009-First week of Augusy 2013) the Unit of Tasfusional Medicine has carried out the treatment with platelet gel of 140 patients (66 females and 74 males) who shared the osteoarticular pain. The treated diseases are reported in Table 1. The age ranged from 26 to 86 years old, with an average of 58 years which was slightly different between genders (mean age for females: 62 years old, mean age for males 53 years old). It was not set up a control group to test treatment versus placebo.

Patients were enrolled using interviewed protocol and scheduled for infiltration ultrasound-echo guided before and after gel platelet infiltration. University hospital Ethics Committee approved the study protocol, and each participant signed an approved informed consent form.

At enrolment, the patients were interviewed to obtain clinical and pharmacological information; blood sampling was performed to acquire the suitability for pre-deposit of whole blood (Hb ≥ 12 g/dl, platelet count > 200.000/μl,) and to assess the healthy state (clinical chemistry tests and virological screening to hepatotropic viruses and HIV).

The criteria for exclusion from the study protocol were:

- Platelet count less than 200,000 plts/L.
- Hemoglobin count less than 11gr/dl.
- Cardiac disease and clinical evaluation unfavorable to the autologous blood donation.
- Vascular access not suitable to pre-deposit autologous blood donation.
- Inability to suspend antiplatelet and/or anticoagulant therapy.
- Previous infiltration or surgical treatment in the places of the injury.

### Materials

Informed consent was obtained from the suitable patients, as well as 350 ml of predeposited autologous whole blood that was collected in quadruple Fresenius Kabi bag.

The production of platelet gel from blood components was performed at the laboratory of Transfusion Medicine of University Hospital “Paolo Giaccone”. Whole blood was centrifugated and separated in order to obtain (through decomposer automatic Compomat G4 Fresenius HemoCare Italy) concentrated platelet and platelet-free plasma (PPP). From the PPP sample, after rapid freezing (− 40°C) followed by a slow thawing (12 hours), cryoprecipitate was collected. Thrombin has been prepared, adding 20 ml of calcium to the supernatant (PPP suite of cryoprecipitate) under a laminar flow hood. The “home made” platelet gel was dispensed into three aliquots containing 8–10 ml platelet concentrate and cryoprecipitate and three aliquots, of equal volume, containing autologous thrombin. The compounds were validated, stored at −20°C and used within one year from date of manufacture.

### Clinical protocol

The study protocol is divided into five different steps: 1) first visit to assess the suitability of the patient to the procedure, 2) second visit in order to obtain the pre-deposit of autologous blood donation 3) production of platelet gel 4) echo ultrasound-guided infiltration at the site of injury 5) clinical-instrumental evaluation of infiltration at different defined time.

The ultrasound-echo guided infiltration of platelet gel was performed by the medical team at specialized orthopedic structure (Medical Center Mantia-CMM) collaborating with the University Hospital.

At the time of infiltration, the platelet gel was activated into a common 20 ml sterile syringe mixing the lysate and platelet thrombin at the ratio of 2:1. Once ready the platelet gel was injected by ultrasound-echo guided infiltration (Esaote My lab) keeping a perpendicular direction to site of the lesion. The amount of platelet lysate infiltrated was approximately 8–10 ml in volume. At the end of treatment, the patient rested in immobility for at least 48 hours.

The assessment on the evolution of the lesion was examined in three time points:

- T0: before treatment.
- T1: after 8 days from infiltration for lesion due to muscle damage, after 15 days for ligament and tendon injuries-after 30 days for treatment of the large joints.
- T2: more than 60 days after infiltration.

During these phases test for pain estimation (VAS), test for joint flexibility (ROM scale) were administered and echo-ultrasound evaluation at the site of infiltration was performed.

With regard to the assessment of pain, each patient was asked to conduct a self-assessment using the VAS (Visual Analog Scale) test.

The VAS test is a continuous scale comprised of a horizontal (HVAS) or vertical (VVAS) line, usually 10centimeters (100 mm) in length, anchored by verbal descriptors for each symptom intensity: the scale is anchored by “no pain” (score of 0) and “pain as bad as it could be” or “worst imaginable pain” (score of 10-100-mm scale-).

The pain response was considered significant when the reduction was at least 50% of the score of the VAS scale a T1 (at a distance of 8 days from the first infiltration for muscle damage, at a distance of 15 days for those ligament and tendon and at a distance of 30 days for large joints).

To estimate the degree of joint flexibility, the medical team used the ROM (Range Of Motion) scale which allows evaluation of the degrees of freedom allowed by a specific articulation. The ROM is usually measured by the number of degrees achieved by a body segment from the starting position to the final position, along its full range of motion and is calculated using a protractor.

Based on ROM assessment is possible to identify three levels: 1)AROM: active range of movement, 2) AAROM: active assisted range of motion; 3) PROM: passive range of motion.

With respect to the functional/joint limitation and the resolution at the injured site after platelet gel application, we chose to use a three points score:

- Score 1: no changed condition from the beginning of treatment.
- Score 2: increase joint ability to perform task but not fully “restitutio ad integrum”.
- Score 3: Complete functional recovery.

The reaction over time was determined in three types of responses:

- No response (NR): permanence of pain and functional limitation and no echo-ultrasonographic evidence of tissue repair.
- Partial response (PR): reduction of pain and/or functional limitation and no or little evidence of ultrasound tissue repair.
- Complete response (CR): important reduction of pain and complete restoration of function with clear ultrasound evidence of tissue repair.

Based on the response to treatment, the medical team evaluated additional indication for further infiltration and established the subsequent time intervals to follow.

### Data analysis

The data collected were represented as mean value. The results of the clinical responses were represented as absolute and relative frequencies (%).
